# An unexpected oxidation: NaK_5_Cl_2_(S_2_O_6_)_2_ revisited

**DOI:** 10.1107/S2056989017000494

**Published:** 2017-01-13

**Authors:** William T. A. Harrison, M. John Plater

**Affiliations:** a Department of Chemistry, University of Aberdeen, Meston Walk, Aberdeen AB24 3UE, Scotland

**Keywords:** crystal structure, di­thio­nate, super-cell, redetermination

## Abstract

The title compound, NaK_5_Cl_2_(S_2_O_6_)_2_ [systematic name: sodium penta­potassium dichloride bis­(di­thio­nate)], arose as an unexpected product from an organic synthesis that used di­thio­nite (S_2_O_4_^2−^) ions as a reducing agent to destroy excess permanganate ions. Compared to the previous study [Stanley (1953 ▸). *Acta Cryst.* **6**, 187–196], the present tetra­gonal structure exhibits a root 2*a* × root 2*a* × *c* super-cell due to subtle changes in the orientations of the di­thio­nate anions. The structure can be visualized as a three-dimensional framework of [001] columns of alternating *trans*-NaO_4_Cl_2_ and KO_4_Cl_2_ octa­hedra cross-linked by the di­thio­nate ions with the inter­stices occupied by KO_6_Cl_2_ polyhedra to generate a densely packed three-dimensional framework. The asymmetric unit comprises two sodium ions (site symmetries 4 and -4, four potassium ions (site symmetries = -4, 4, 1 and 1), three chloride ions (site symmetries = 4, 4 and 2) and two half-di­thio­nate ions (all atoms on general positions). Both di­thio­nate ions are completed by crystallographic inversion symmetry. The crystal chosen for data collection was found to be rotationally twinned by 180° about the [100] axis in reciprocal space with a 0.6298 (13):0.3702 (13) domain ratio.

## Abstract 2

The redetermined structure of NaK_5_Cl_2_(S_2_O_6_)_2_ shows a super-cell, which accommodates subtle changes in the orientation of the O atoms of the di­thio­nate groups compared to the previously reported structure.

## Chemical context   

As well as their large-scale industrial use in reducing and solublizing vat dyes such as indigo (Božič & Kokol, 2008 ▸), di­thio­nites containing the S_2_O_4_^2−^ anion (sulfur oxidation state = +3) have long found use as moderately strong reducing agents in organic synthesis (De Vries & Kellogg, 1980 ▸, and references therein). The title mixed-cation, mixed-anion compound, NaK_5_Cl_2_(S_2_O_6_)_2_ (I), containing S_2_O_6_^2−^ di­thio­nate ions (sulfur oxidation state = +5), arose as a completely unexpected side product from an attempt to oxidize hexa­methyl benzene to mellitic acid as a precursor of synthetic mellite (Plater & Harrison, 2015 ▸): sodium di­thio­nite was added to the reaction to destroy excess permanganate ions (as KMnO_4_) and the source of the chloride ions was added HCl. To our slight surprise, the structure of the title compound, along with that of the non-isostructural Na_2_K_4_Cl_2_(S_2_O_6_)_2_, was established over 60 years ago (Stanley, 1953 ▸). This re-determination presents a superstructure of the earlier reported structure, which arises from subtle orientational changes for the di­thio­nate anions.

## Structural commentary   

Compound (I) comprises two sodium ions (Na1 site symmetry = 4, Na2 site symmetry = 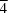), four potassium ions (site symmetries = 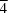, 4, 1 and 1 for K1, K2, K3 and K4, respectively), three chloride ions (Cl1 and Cl2 with site symmetry 4, Cl3 with site symmetry 2) and two half-di­thio­nate ions (all atoms on general positions) in the asymmetric unit. Selected geometrical data are given in Table 1 ▸. Both S_2_O_6_^2−^ di­thio­nate ions are completed by crystallographic inversion symmetry at the mid-points of their S—S bonds [S1—S1^i^ = 2.1227 (9), S2—S2^xiii^ = 2.1176 (9) Å; see Table 1 ▸ for symmetry codes] and both exhibit almost ideal staggered conformations about their S—S bonds. The mean S—O bond length (both unique ions) is 1.45 Å and the narrow spread of individual S—O bond lengths from 1.4465 (11) to 1.4526 (13) Å indicates that the negative charges of the anion are delocalized over the three O atoms attached to each S atom (*i.e*.: we cannot identify localized S=O double bonds and S—O single bonds). In terms of the orientation of the di­thio­nate ions in the unit cell, the S1—O1 bond deviates from the (001) plane by 12.5° and the S2—O5 bond deviates by 10.6° (*vide infra*).

**Figure 1:**
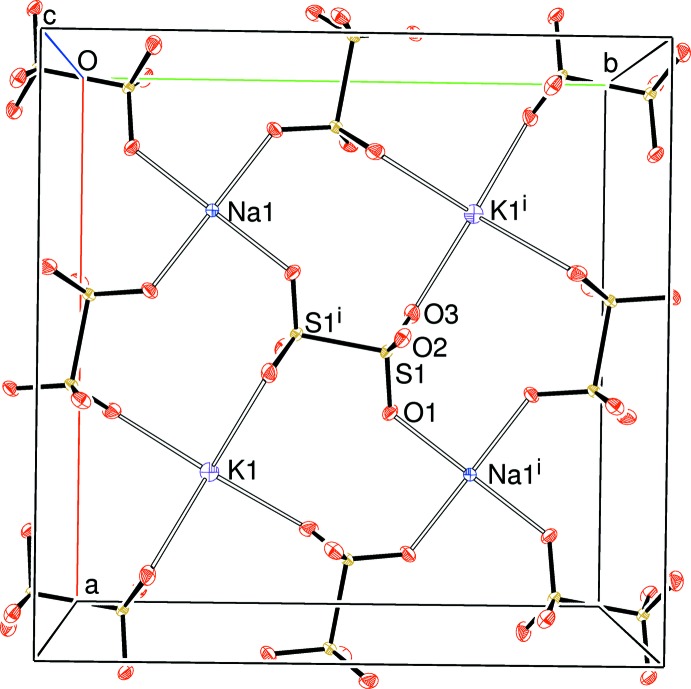
View down [001] of a slice (−0.15 ≤ *z* ≤ 0.15) of the structure of (I). Displacement ellipsoids are displayed at the 50% probability level. [Symmetry code: (i) 1 − *x*, 1 − *y*, −*z*.] Note that Na1 lies on a fourfold axis and K1 has 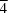 site symmetry.

**Figure 2:**
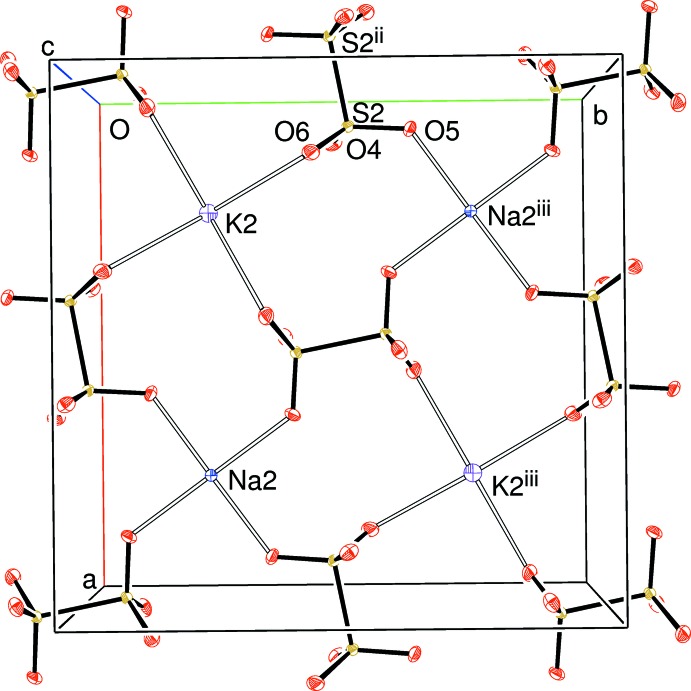
View down [001] of a slice (0.35 ≤ *z* ≤ 0.65) of the structure of (I). Displacement ellipsoids are displayed at the 50% probability level. [Symmetry codes: (ii) −*x*, 1 − *y*, 1 − *z*; (iii) 1 − *x*, 1 − *y*, 1 − *z*.] Note that K2 lies on a fourfold axis and Na2 has 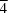 site symmetry.

**Figure 3:**
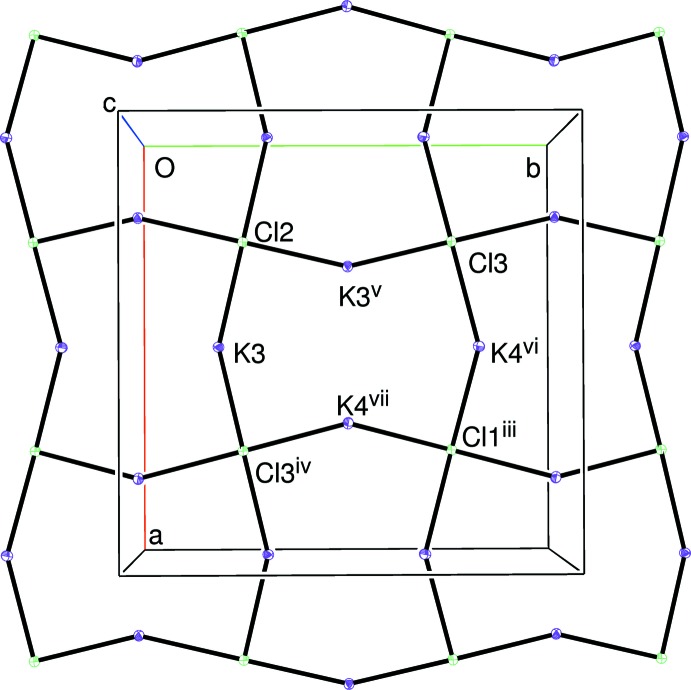
View down [001] of a slice (0.25 ≤ *z* ≤ 0.35) of the structure of (I). Displacement ellipsoids are displayed at the 50% probability level. [Symmetry codes: (iii) 1 − *x*, 1 − *y*, 1 − *z*; (iv) 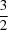 − *y*, *x*, *z*; (v) 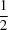 − *y*, *x*, *z*; (vi) 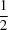 + *y*, 1 − *x*, 1 − *z*; (vii) 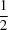 + *x*, 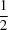 + *y*, 1 − *z*.] Note that Cl1 and Cl2 lie on fourfold axes and Cl3 lies on a twofold axis. The chloride ions link to the sodium and potassium ions shown in Figs. 1 ▸ and 2 ▸ to generate infinite stacks of alternating NaO_4_Cl_2_ and KO_4_Cl_2_ octa­hedra.

The packing for (I) can be described in terms of *pseudo* layers lying perpendicular to the *c*-axis direction of the tetra­gonal unit cell. At *z* ∼ 0 and 1, the S1/O1/O2/O3 di­thio­nate ion and the Na1 and K1 cations reside (Fig. 1 ▸); at *z* ∼1/2, are to be found the S2/O4/O5/O6 di­thio­nate ion and Na2 and K2 (Fig. 2 ▸). Between them, at *z* ∼ 1/4 and 3/4, are K3, K4 and the three chloride ions, which form a distorted square grid (Fig. 3 ▸).

**Figure 4:**
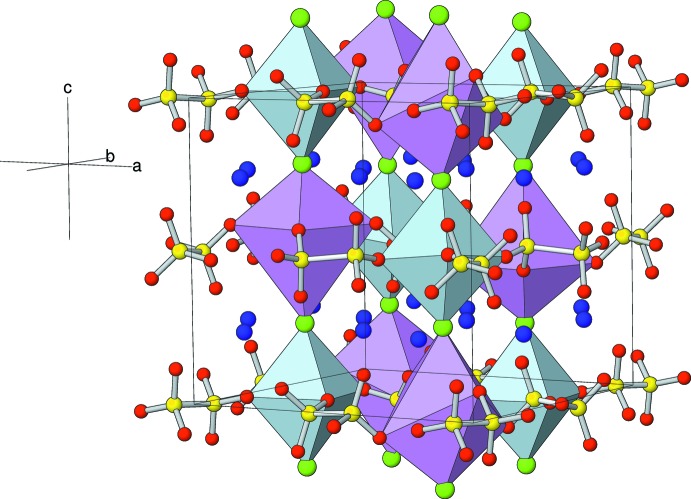
Polyhedral view of the structure of (I), showing the [001] chains of NaO_4_Cl_2_ octa­hedra (blue) and KO_4_Cl_2_ (K1 and K2) octa­hedra (lilac) cross-linked by the di­thio­nate groups. Atoms K3 and K4 are shown as purple spheres.

The extended structure of (I) can be visualized (Fig. 4 ▸) as [001] chains of alternating *trans*-NaO_4_Cl_2_ and KO_4_Cl_2_ octa­hedra linked *via* their chloride ions and cross-linked by the di­thio­nate groups. There are two distinct chains: the Na1 and K2 species and their linking chloride ions (Cl1 and Cl2) lie on the fourfold axes at (1/4, 1/4, *z*) and (3/4, 3/4, *z*), whereas Na2 and K1 (both site symmetry 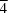) are connected by Cl3, which lies on the (1/4, 3/4, *z*) twofold axis and its symmetry-generated clone at (3/4, 1/4, *z*). As expected, the Na—O bonds (mean = 2.34 Å) are much shorter than the K—O bonds (mean = 2.82 Å). In terms of bond angles, the sodium-centred octa­hedra are almost regular [spread of *cis* and *trans* bond angles = 87.94 (4)–92.06 (4) and 175.89 (8)–180°, respectively, for Na1 and 86.02 (3)–93.98 (3) and 172.03 (5)–180°, respectively, for Na2] but the potassium-centred moieties are grossly distorted with ranges of *cis* and *trans* angles of 71.75 (2)–108.25 (2) and 143.50 (4)–180°, respectively, for K1 and 74.91 (2)–105.09 (2) and 149.82 (5)–180°, respectively, for K2.

The structure of (I) is completed by the K3 and K4 potassium ions, which occupy inter­stices in the framework described in the preceding paragraph. The K3 coordination polyhedron approximates to an extremely distorted KO_6_Cl_2_ square anti-prism. The coordination for K4 is slightly ambiguous, with six shorter K—O bonds [2.8429 (11)–3.0664 (12) Å] and two K—Cl links [3.1168 (5) and 3.1291 (5) Å] forming a squashed and distorted square anti-prism. There are two further K4⋯O close contacts at 3.2273 (12) and 3.3822 (12) Å [the next-nearest K4⋯O separation after these is 4.3935 (13) Å] but given that these K4⋯O contacts are longer than the K4—Cl bonds and have bond valences (Brown & Altermatt, 1985 ▸) of less than 0.05 (Brown, 2002 ▸), we regard them as not significant. The three chloride ions each adopt almost regular ClK_5_Na octa­hedral geometries.

Bond-valence sum (BVS) data (Brown & Altermatt, 1985 ▸) for the cations in (I) indicate that the sodium ions in (I) are considerably ‘overbonded’: BVS(Na1) = 1.46 and BVS(Na2) = 1.45 (expected value = 1.0 valence units). Three of the potassium ions are possibly slightly over-bonded (BVS values for K1, K2 and K3 = 1.16, 1.19 and 1.19, respectively) whereas K4 (BVS = 1.01) achieves its expected valence almost exactly.

The previously-reported structure of NaK_5_Cl_2_(S_2_O_6_)_2_ (Stanley, 1953 ▸) was modelled in space group *P*4/*mnc* [*a*_S_ = 8.5621 (6), *c*_S_ = 11.5288 (6) Å, *V*_S_ = 845.2 Å^3^; S = Stanley], thus it may be seen that the present unit cell is a 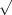
2*a*_S_ × 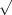
2*a*_S_ × *c*_S_ super-cell of the Stanley structure with doubled volume. The relative dispositions of the sodium, potassium and chloride ions in the Stanley structure are almost the same as in (I); the main difference occurs in the orientation of the di­thio­nate ions with respect to the (001) plane; in the Stanley structure, this species, which is built up from one unique S atom and two unique O atoms, has 2/*m* (*C*_2*h*_) point-group symmetry about the mid-point of the S—S bond with the S atom and one of the O atoms lying on the *z* = 0 mirror plane [compare the deviations from the (001) plane noted above for the S1—O1 and S2—O5 bonds in (I)].

## Database survey   

As already noted, this structure (ICSD reference number 24676) was previously reported by Stanley (1953 ▸). A survey of the Cambridge Structural Database (Groom *et al.*, 2016 ▸) (entries updated to 20 December 2016) revealed 138 crystal structures containing di­thio­nate anions.

## Synthesis and crystallization   

In an attempt to prepare mellitic acid (C_6_H_6_O_12_) as a precursor of synthetic mellite (Plater & Harrison, 2015 ▸), hexa­methyl­benzene (2.0 g, 0.0123 moles) and KMnO_4_ (23.4 g, 0.148 moles, 12 equiv.) were refluxed in water for 24 h (Friedel & Crafts, 1884 ▸): the organic starting material had a tendency to sublime into the condenser. After cooling, the mixture was treated with excess Na_2_S_2_O_4_ to decompose the unreacted permanganate, which turned the solution brown. It was filtered and then treated with conc. HCl to give a pH of 1. After leaving to crystallize, the solid product (1.34 g) was collected by filtration as colourless blocks of (I). Evidently, di­thio­nite has been oxidized by permanganate to di­thio­nate by an unknown pathway and the sodium and potassium cations and chloride ions (from the hydro­chloric acid) present in the mixture serendipitiously combine with the di­thio­nate ions to form (I).

## Refinement   

Crystal data, data collection and structure refinement details are summarized in Table 2 ▸. All the atoms in the asymmetric unit were located by *SHELXT* (Sheldrick, 2015*a* ▸): the potassium cations and chloride anions were distinguished in terms of chemically reasonable environments. The crystal chosen for data collection was found to be rotationally twinned by 180° about the [100] axis in reciprocal space with a 0.6298 (13):0.3702 (13) domain ratio.

## Supplementary Material

Crystal structure: contains datablock(s) I, global. DOI: 10.1107/S2056989017000494/wm5357sup1.cif

Structure factors: contains datablock(s) I. DOI: 10.1107/S2056989017000494/wm5357Isup2.hkl

CCDC reference: 1526611

Additional supporting information:  crystallographic information; 3D view; checkCIF report

## Figures and Tables

**Table 1:** Selected bond lengths (Å)

Na1—O1^i^	2.3375 (13)	K3—Cl2	3.1088 (5)
Na1—Cl1^ii^	2.6942 (13)	K4—O2^viii^	2.8429 (11)
Na1—Cl2	2.7260 (12)	K4—O6^vi^	2.9235 (11)
Na2—O5^iii^	2.3506 (13)	K4—O6^ix^	2.9941 (11)
Na2—Cl3^iv^	2.6986 (5)	K4—O4^x^	3.0284 (12)
K1—O3^v^	2.8262 (12)	K4—O3^xi^	3.0290 (11)
K1—Cl3^iv^	2.9976 (5)	K4—O2^xii^	3.0664 (12)
K2—O6	2.8193 (11)	K4—Cl1	3.1168 (5)
K2—Cl2	2.9746 (8)	K4—Cl3^x^	3.1291 (5)
K2—Cl1	2.9978 (9)	S1—O3	1.4465 (11)
K3—O4^v^	2.7843 (11)	S1—O2	1.4507 (10)
K3—O2^v^	2.8705 (11)	S1—O1	1.4526 (13)
K3—O4^vi^	2.8973 (11)	S1—S1^i^	2.1227 (9)
K3—O3^i^	2.8995 (11)	S2—O6	1.4475 (11)
K3—O1^vii^	2.9148 (11)	S2—O5	1.4505 (13)
K3—O5^iii^	3.0176 (11)	S2—O4	1.4516 (10)
K3—Cl3^iv^	3.0836 (5)	S2—S2^xiii^	2.1176 (9)

Symmetry codes: (i) 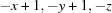; (ii) 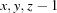; (iii) 
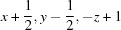
; (iv) 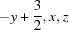; (v) 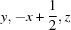; (vi) 
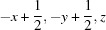
; (vii) 
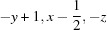
; (viii) 
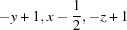
; (ix) 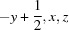; (x) 
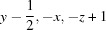
; (xi) 
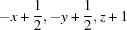
; (xii) 
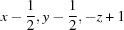
; (xiii) 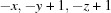.

**Table 2:** Experimental details

Crystal data	
Chemical formula	NaK_5_Cl_2_(S_2_O_6_)_2_
*M*_r_	609.63
Crystal system, space group	Tetragonal, *P*4/*n*
Temperature (K)	100
*a*, *c* (Å)	12.0421 (1), 11.3925 (2)
*V* (Å^3^)	1652.05 (4)
*Z*	4
Radiation type	Mo *K*α
μ (mm^−1^)	2.24
Crystal size (mm)	0.05 × 0.02 × 0.02

Data collection	
Diffractometer	Rigaku Mercury CCD
Absorption correction	Multi-scan (*SADABS*; Sheldrick, 2004 ▸)
*T*_min_, *T*_max_	0.911, 1.000
No. of measured, independent and observed [*I* > 2σ(*I*)] reflections	1910, 1910, 1834
(sin θ/λ)_max_ (Å^−1^)	0.649

Refinement	
*R*[*F* ^2^ > 2σ(*F* ^2^)], *wR*(*F* ^2^), *S*	0.015, 0.043, 1.06
No. of reflections	1910
No. of parameters	113
Δρ_max_, Δρ_min_ (e Å^−3^)	0.35, −0.35

Computer programs: *CrystalClear* (Rigaku, 2010 ▸), *SHELXT* (Sheldrick, 2015*a* ▸), *SHELXL2014* (Sheldrick, 2015*b* ▸), *ORTEP-3 for Windows* (Farrugia, 2012 ▸) and *ATOMS* (Dowty, 1999 ▸).

## supplementary crystallographic information

### Crystal data

**Table:**

NaK_5_Cl_2_(S_2_O_6_)_2_	*D*_x_ = 2.451 Mg m^−^^3^
*M**_r_* = 609.63	Mo *K*α radiation, λ = 0.71073 Å
Tetragonal, *P*4/*n*	Cell parameters from 23162 reflections
*a* = 12.0421 (1) Å	θ = 2.3–27.5°
*c* = 11.3925 (2) Å	µ = 2.24 mm^−^^1^
*V* = 1652.05 (4) Å^3^	*T* = 100 K
*Z* = 4	Block, colourless
*F*(000) = 1200	0.05 × 0.02 × 0.02 mm

### Data collection

**Table:**

Rigaku Mercury CCD diffractometer	1834 reflections with *I* > 2σ(*I*)
ω scans	θ_max_ = 27.5°, θ_min_ = 1.8°
Absorption correction: multi-scan (SADABS; Sheldrick, 2004)	*h* = −10→11
*T*_min_ = 0.911, *T*_max_ = 1.000	*k* = −15→15
1910 measured reflections	*l* = −14→14
1910 independent reflections	

### Refinement

**Table:**

Refinement on *F*^2^	0 restraints
Least-squares matrix: full	Primary atom site location: structure-invariant direct methods
*R*[*F*^2^ > 2σ(*F*^2^)] = 0.015	*w* = 1/[σ^2^(*F*_o_^2^) + (0.0242*P*)^2^ + 0.4886*P*] where *P* = (*F*_o_^2^ + 2*F*_c_^2^)/3
*wR*(*F*^2^) = 0.043	(Δ/σ)_max_ < 0.001
*S* = 1.06	Δρ_max_ = 0.35 e Å^−^^3^
1910 reflections	Δρ_min_ = −0.35 e Å^−^^3^
113 parameters	

### Special details

**Table:**

Geometry. All esds (except the esd in the dihedral angle between two l.s. planes) are estimated using the full covariance matrix. The cell esds are taken into account individually in the estimation of esds in distances, angles and torsion angles; correlations between esds in cell parameters are only used when they are defined by crystal symmetry. An approximate (isotropic) treatment of cell esds is used for estimating esds involving l.s. planes.
Refinement. Refined as a 2-component twin.

### Fractional atomic coordinates and isotropic or equivalent isotropic displacement parameters (Å^2^)

**Table:**

	*x*	*y*	*z*	*U*_iso_*/*U*_eq_	
Na1	0.2500	0.2500	0.01268 (9)	0.0094 (3)	
Na2	0.7500	0.2500	0.5000	0.0084 (3)	
K1	0.7500	0.2500	0.0000	0.01621 (19)	
K2	0.2500	0.2500	0.51306 (5)	0.01506 (18)	
K3	0.50130 (4)	0.19113 (2)	0.24632 (3)	0.01042 (8)	
K4	0.18440 (2)	0.00129 (4)	0.74572 (3)	0.01187 (8)	
S1	0.51535 (4)	0.58649 (4)	0.00758 (3)	0.00962 (10)	
O1	0.63276 (11)	0.59544 (11)	−0.02005 (10)	0.0160 (3)	
O2	0.48827 (9)	0.61202 (9)	0.12870 (9)	0.0163 (2)	
O3	0.44243 (10)	0.63754 (9)	−0.07768 (9)	0.0190 (2)	
S2	0.08586 (4)	0.51687 (4)	0.49086 (3)	0.00872 (10)	
O4	0.10996 (9)	0.48723 (9)	0.37002 (9)	0.0152 (2)	
O5	0.09283 (11)	0.63505 (10)	0.51433 (9)	0.0142 (3)	
O6	0.13966 (9)	0.44729 (9)	0.57748 (9)	0.0169 (2)	
Cl1	0.2500	0.2500	0.77619 (6)	0.00944 (14)	
Cl2	0.2500	0.2500	0.25196 (5)	0.00958 (16)	
Cl3	0.2500	0.7500	0.26312 (4)	0.00951 (13)	

### Atomic displacement parameters (Å^2^)

**Table:**

	*U*^11^	*U*^22^	*U*^33^	*U*^12^	*U*^13^	*U*^23^
K1	0.0200 (3)	0.0200 (3)	0.0086 (3)	0.000	0.000	0.000
K2	0.0183 (3)	0.0183 (3)	0.0086 (3)	0.000	0.000	0.000
K3	0.00940 (15)	0.01049 (13)	0.01136 (15)	0.00021 (17)	0.00028 (9)	−0.00154 (12)
K4	0.01177 (13)	0.00978 (15)	0.01406 (15)	−0.00037 (17)	−0.00130 (13)	0.00016 (10)
Na1	0.0094 (5)	0.0094 (5)	0.0095 (5)	0.000	0.000	0.000
Na2	0.0080 (5)	0.0080 (5)	0.0090 (5)	0.000	0.000	0.000
S1	0.0104 (2)	0.0092 (2)	0.00920 (16)	−0.00190 (19)	0.00093 (11)	−0.00018 (12)
O1	0.0137 (7)	0.0134 (6)	0.0208 (5)	−0.0041 (6)	0.0057 (4)	−0.0037 (5)
O2	0.0201 (6)	0.0156 (5)	0.0131 (5)	−0.0047 (5)	0.0054 (5)	−0.0053 (4)
O3	0.0211 (6)	0.0137 (5)	0.0223 (6)	−0.0004 (5)	−0.0074 (5)	0.0047 (5)
S2	0.0080 (2)	0.0088 (2)	0.00928 (16)	−0.00075 (18)	0.00053 (12)	−0.00015 (12)
O4	0.0139 (5)	0.0188 (6)	0.0129 (5)	−0.0036 (5)	0.0053 (4)	−0.0044 (4)
O5	0.0134 (7)	0.0091 (6)	0.0200 (5)	−0.0031 (5)	0.0024 (4)	−0.0026 (4)
O6	0.0129 (5)	0.0176 (6)	0.0202 (5)	−0.0001 (5)	−0.0040 (4)	0.0061 (5)
Cl1	0.0100 (2)	0.0100 (2)	0.0084 (3)	0.000	0.000	0.000
Cl2	0.0099 (2)	0.0099 (2)	0.0090 (3)	0.000	0.000	0.000
Cl3	0.0107 (3)	0.0093 (2)	0.0085 (2)	0.00012 (15)	0.000	0.000

### Geometric parameters (Å, º)

**Table:**

Na1—O1^i^	2.3375 (13)	K4—O5^xiv^	3.2273 (12)
Na1—O1^ii^	2.3375 (13)	K4—O1^xvi^	3.3822 (12)
Na1—O1^iii^	2.3375 (13)	S1—O3	1.4465 (11)
Na1—O1^iv^	2.3375 (13)	S1—O2	1.4507 (10)
Na1—Cl1^v^	2.6942 (13)	S1—O1	1.4526 (13)
Na1—Cl2	2.7260 (12)	S1—S1^i^	2.1227 (9)
Na2—O5^vi^	2.3506 (13)	O1—Na1^i^	2.3375 (13)
Na2—O5^vii^	2.3506 (13)	O1—K3^xvii^	2.9148 (11)
Na2—O5^viii^	2.3506 (13)	O1—K4^xviii^	3.3822 (12)
Na2—O5^ix^	2.3506 (13)	O2—K4^xix^	2.8429 (11)
Na2—Cl3^ix^	2.6986 (5)	O2—K3^xi^	2.8705 (11)
Na2—Cl3^vii^	2.6986 (5)	O2—K4^xviii^	3.0664 (12)
K1—O3^viii^	2.8262 (12)	O3—K1^i^	2.8262 (12)
K1—O3^ix^	2.8262 (12)	O3—K3^i^	2.8995 (11)
K1—O3^x^	2.8262 (12)	O3—K4^xx^	3.0290 (11)
K1—O3^i^	2.8262 (12)	S2—O6	1.4475 (11)
K1—Cl3^ix^	2.9976 (5)	S2—O5	1.4505 (13)
K1—Cl3^i^	2.9976 (5)	S2—O4	1.4516 (10)
K2—O6	2.8193 (11)	S2—S2^xxi^	2.1176 (9)
K2—O6^viii^	2.8193 (12)	O4—K3^xi^	2.7843 (11)
K2—O6^xi^	2.8193 (12)	O4—K3^xii^	2.8973 (11)
K2—O6^xii^	2.8193 (11)	O4—K4^xxii^	3.0284 (12)
K2—Cl2	2.9746 (8)	O5—Na2^vii^	2.3506 (13)
K2—Cl1	2.9978 (9)	O5—K3^xxiii^	3.0176 (11)
K3—O4^viii^	2.7843 (11)	O5—K4^xxii^	3.2273 (12)
K3—O2^viii^	2.8705 (11)	O6—K4^xii^	2.9235 (11)
K3—O4^xii^	2.8973 (11)	O6—K4^viii^	2.9941 (11)
K3—O3^i^	2.8995 (11)	Cl1—Na1^xxiv^	2.6943 (13)
K3—O1^iii^	2.9148 (11)	Cl1—K4^xii^	3.1168 (5)
K3—O5^vi^	3.0176 (11)	Cl1—K4^viii^	3.1168 (5)
K3—Cl3^ix^	3.0836 (5)	Cl1—K4^xi^	3.1168 (5)
K3—Cl2	3.1088 (5)	Cl2—K3^viii^	3.1088 (5)
K4—O2^xiii^	2.8429 (11)	Cl2—K3^xi^	3.1088 (5)
K4—O6^xii^	2.9235 (11)	Cl2—K3^xii^	3.1088 (5)
K4—O6^xi^	2.9941 (11)	Cl3—Na2^vii^	2.6987 (5)
K4—O4^xiv^	3.0284 (12)	Cl3—K1^i^	2.9976 (5)
K4—O3^xv^	3.0290 (11)	Cl3—K3^xi^	3.0835 (5)
K4—O2^xvi^	3.0664 (12)	Cl3—K3^xxv^	3.0835 (5)
K4—Cl1	3.1168 (5)	Cl3—K4^xxii^	3.1291 (5)
K4—Cl3^xiv^	3.1291 (5)	Cl3—K4^xix^	3.1291 (5)
			
O1^i^—Na1—O1^ii^	175.89 (8)	O6^xii^—K4—Cl3^xiv^	90.04 (2)
O1^i^—Na1—O1^iii^	89.926 (3)	O6^xi^—K4—Cl3^xiv^	130.64 (2)
O1^ii^—Na1—O1^iii^	89.926 (3)	O4^xiv^—K4—Cl3^xiv^	75.88 (2)
O1^i^—Na1—O1^iv^	89.926 (3)	O3^xv^—K4—Cl3^xiv^	67.35 (2)
O1^ii^—Na1—O1^iv^	89.926 (3)	O2^xvi^—K4—Cl3^xiv^	129.05 (2)
O1^iii^—Na1—O1^iv^	175.89 (8)	Cl1—K4—Cl3^xiv^	150.327 (9)
O1^i^—Na1—Cl1^v^	92.06 (4)	O2^xiii^—K4—O5^xiv^	126.97 (3)
O1^ii^—Na1—Cl1^v^	92.06 (4)	O6^xii^—K4—O5^xiv^	66.79 (3)
O1^iii^—Na1—Cl1^v^	92.06 (4)	O6^xi^—K4—O5^xiv^	60.67 (3)
O1^iv^—Na1—Cl1^v^	92.06 (4)	O4^xiv^—K4—O5^xiv^	45.62 (3)
O1^i^—Na1—Cl2	87.94 (4)	O3^xv^—K4—O5^xiv^	108.85 (3)
O1^ii^—Na1—Cl2	87.94 (4)	O2^xvi^—K4—O5^xiv^	116.05 (3)
O1^iii^—Na1—Cl2	87.94 (4)	Cl1—K4—O5^xiv^	119.10 (3)
O1^iv^—Na1—Cl2	87.94 (4)	Cl3^xiv^—K4—O5^xiv^	71.17 (3)
Cl1^v^—Na1—Cl2	180.0	O2^xiii^—K4—O1^xvi^	73.26 (3)
O5^vi^—Na2—O5^vii^	172.03 (5)	O6^xii^—K4—O1^xvi^	131.92 (3)
O5^vi^—Na2—O5^viii^	90.277 (4)	O6^xi^—K4—O1^xvi^	107.92 (3)
O5^vii^—Na2—O5^viii^	90.277 (4)	O4^xiv^—K4—O1^xvi^	113.99 (3)
O5^vi^—Na2—O5^ix^	90.277 (4)	O3^xv^—K4—O1^xvi^	58.57 (3)
O5^vii^—Na2—O5^ix^	90.277 (4)	O2^xvi^—K4—O1^xvi^	44.07 (3)
O5^viii^—Na2—O5^ix^	172.03 (5)	Cl1—K4—O1^xvi^	67.76 (3)
O5^vi^—Na2—Cl3^ix^	86.02 (3)	Cl3^xiv^—K4—O1^xvi^	113.60 (2)
O5^vii^—Na2—Cl3^ix^	86.02 (3)	O5^xiv^—K4—O1^xvi^	158.77 (2)
O5^viii^—Na2—Cl3^ix^	93.98 (3)	O3—S1—O2	114.34 (7)
O5^ix^—Na2—Cl3^ix^	93.98 (3)	O3—S1—O1	114.44 (7)
O5^vi^—Na2—Cl3^vii^	93.98 (3)	O2—S1—O1	114.16 (7)
O5^vii^—Na2—Cl3^vii^	93.98 (3)	O3—S1—S1^i^	104.87 (6)
O5^viii^—Na2—Cl3^vii^	86.02 (3)	O2—S1—S1^i^	104.25 (5)
O5^ix^—Na2—Cl3^vii^	86.02 (3)	O1—S1—S1^i^	102.99 (6)
Cl3^ix^—Na2—Cl3^vii^	180.0	S1—O1—Na1^i^	129.73 (8)
O3^viii^—K1—O3^ix^	143.50 (4)	S1—O1—K3^xvii^	113.37 (6)
O3^viii^—K1—O3^x^	95.627 (13)	Na1^i^—O1—K3^xvii^	101.79 (5)
O3^ix^—K1—O3^x^	95.627 (13)	S1—O1—K4^xviii^	87.35 (5)
O3^viii^—K1—O3^i^	95.627 (13)	Na1^i^—O1—K4^xviii^	97.05 (5)
O3^ix^—K1—O3^i^	95.627 (13)	K3^xvii^—O1—K4^xviii^	129.71 (5)
O3^x^—K1—O3^i^	143.50 (4)	S1—O2—K4^xix^	130.46 (6)
O3^viii^—K1—Cl3^ix^	108.25 (2)	S1—O2—K3^xi^	120.98 (6)
O3^ix^—K1—Cl3^ix^	108.25 (2)	K4^xix^—O2—K3^xi^	101.95 (3)
O3^x^—K1—Cl3^ix^	71.75 (2)	S1—O2—K4^xviii^	100.28 (6)
O3^i^—K1—Cl3^ix^	71.75 (2)	K4^xix^—O2—K4^xviii^	95.61 (3)
O3^viii^—K1—Cl3^i^	71.75 (2)	K3^xi^—O2—K4^xviii^	99.20 (3)
O3^ix^—K1—Cl3^i^	71.75 (2)	S1—O3—K1^i^	119.41 (6)
O3^x^—K1—Cl3^i^	108.25 (2)	S1—O3—K3^i^	127.26 (6)
O3^i^—K1—Cl3^i^	108.25 (2)	K1^i^—O3—K3^i^	93.33 (3)
Cl3^ix^—K1—Cl3^i^	180.0	S1—O3—K4^xx^	121.17 (6)
O6—K2—O6^viii^	86.115 (12)	K1^i^—O3—K4^xx^	93.38 (3)
O6—K2—O6^xi^	86.115 (12)	K3^i^—O3—K4^xx^	94.04 (3)
O6^viii^—K2—O6^xi^	149.82 (5)	O6—S2—O5	114.61 (7)
O6—K2—O6^xii^	149.82 (5)	O6—S2—O4	114.50 (7)
O6^viii^—K2—O6^xii^	86.115 (12)	O5—S2—O4	113.86 (7)
O6^xi^—K2—O6^xii^	86.115 (12)	O6—S2—S2^xxi^	105.01 (5)
O6—K2—Cl2	105.09 (2)	O5—S2—S2^xxi^	103.10 (6)
O6^viii^—K2—Cl2	105.09 (2)	O4—S2—S2^xxi^	103.96 (5)
O6^xi^—K2—Cl2	105.09 (2)	S2—O5—Na2^vii^	127.70 (8)
O6^xii^—K2—Cl2	105.09 (2)	S2—O5—K3^xxiii^	111.41 (6)
O6—K2—Cl1	74.91 (2)	Na2^vii^—O5—K3^xxiii^	103.01 (4)
O6^viii^—K2—Cl1	74.91 (2)	S2—O5—K4^xxii^	89.49 (5)
O6^xi^—K2—Cl1	74.91 (2)	Na2^vii^—O5—K4^xxii^	96.35 (4)
O6^xii^—K2—Cl1	74.91 (2)	K3^xxiii^—O5—K4^xxii^	131.31 (5)
Cl2—K2—Cl1	180.0	S2—O6—K2	121.42 (6)
O4^viii^—K3—O2^viii^	155.78 (4)	S2—O6—K4^xii^	130.44 (6)
O4^viii^—K3—O4^xii^	111.31 (5)	K2—O6—K4^xii^	90.43 (3)
O2^viii^—K3—O4^xii^	83.34 (3)	S2—O6—K4^viii^	119.60 (6)
O4^viii^—K3—O3^i^	74.78 (3)	K2—O6—K4^viii^	89.00 (3)
O2^viii^—K3—O3^i^	96.76 (3)	K4^xii^—O6—K4^viii^	95.50 (3)
O4^xii^—K3—O3^i^	163.31 (3)	Na1^xxiv^—Cl1—K2	180.0
O4^viii^—K3—O1^iii^	129.10 (4)	Na1^xxiv^—Cl1—K4	96.394 (14)
O2^viii^—K3—O1^iii^	65.88 (3)	K2—Cl1—K4	83.606 (14)
O4^xii^—K3—O1^iii^	93.82 (3)	Na1^xxiv^—Cl1—K4^xii^	96.394 (14)
O3^i^—K3—O1^iii^	71.28 (3)	K2—Cl1—K4^xii^	83.606 (14)
O4^viii^—K3—O5^vi^	75.96 (3)	K4—Cl1—K4^xii^	167.21 (3)
O2^viii^—K3—O5^vi^	94.81 (3)	Na1^xxiv^—Cl1—K4^viii^	96.394 (14)
O4^xii^—K3—O5^vi^	64.12 (3)	K2—Cl1—K4^viii^	83.606 (14)
O3^i^—K3—O5^vi^	132.27 (4)	K4—Cl1—K4^viii^	89.290 (3)
O1^iii^—K3—O5^vi^	153.02 (3)	K4^xii^—Cl1—K4^viii^	89.289 (3)
O4^viii^—K3—Cl3^ix^	80.21 (2)	Na1^xxiv^—Cl1—K4^xi^	96.394 (14)
O2^viii^—K3—Cl3^ix^	75.58 (2)	K2—Cl1—K4^xi^	83.606 (14)
O4^xii^—K3—Cl3^ix^	126.11 (2)	K4—Cl1—K4^xi^	89.289 (3)
O3^i^—K3—Cl3^ix^	69.55 (2)	K4^xii^—Cl1—K4^xi^	89.289 (3)
O1^iii^—K3—Cl3^ix^	119.90 (3)	K4^viii^—Cl1—K4^xi^	167.21 (3)
O5^vi^—K3—Cl3^ix^	68.94 (3)	Na1—Cl2—K2	180.0
O4^viii^—K3—Cl2	74.58 (2)	Na1—Cl2—K3	88.815 (12)
O2^viii^—K3—Cl2	129.30 (2)	K2—Cl2—K3	91.185 (12)
O4^xii^—K3—Cl2	73.07 (2)	Na1—Cl2—K3^viii^	88.814 (12)
O3^i^—K3—Cl2	94.52 (3)	K2—Cl2—K3^viii^	91.186 (12)
O1^iii^—K3—Cl2	71.59 (3)	K3—Cl2—K3^viii^	89.976 (1)
O5^vi^—K3—Cl2	112.82 (3)	Na1—Cl2—K3^xi^	88.815 (12)
Cl3^ix^—K3—Cl2	153.089 (9)	K2—Cl2—K3^xi^	91.185 (12)
O2^xiii^—K4—O6^xii^	73.54 (3)	K3—Cl2—K3^xi^	89.975 (1)
O2^xiii^—K4—O6^xi^	144.06 (3)	K3^viii^—Cl2—K3^xi^	177.63 (2)
O6^xii^—K4—O6^xi^	81.16 (4)	Na1—Cl2—K3^xii^	88.814 (12)
O2^xiii^—K4—O4^xiv^	150.56 (4)	K2—Cl2—K3^xii^	91.186 (12)
O6^xii^—K4—O4^xiv^	112.11 (3)	K3—Cl2—K3^xii^	177.63 (2)
O6^xi^—K4—O4^xiv^	63.40 (3)	K3^viii^—Cl2—K3^xii^	89.975 (1)
O2^xiii^—K4—O3^xv^	94.02 (3)	K3^xi^—Cl2—K3^xii^	89.975 (1)
O6^xii^—K4—O3^xv^	156.61 (4)	Na2^vii^—Cl3—K1^i^	180.0
O6^xi^—K4—O3^xv^	117.73 (3)	Na2^vii^—Cl3—K3^xi^	93.559 (11)
O4^xiv^—K4—O3^xv^	69.49 (3)	K1^i^—Cl3—K3^xi^	86.441 (11)
O2^xiii^—K4—O2^xvi^	116.96 (4)	Na2^vii^—Cl3—K3^xxv^	93.559 (11)
O6^xii^—K4—O2^xvi^	140.54 (3)	K1^i^—Cl3—K3^xxv^	86.441 (11)
O6^xi^—K4—O2^xvi^	69.44 (3)	K3^xi^—Cl3—K3^xxv^	172.88 (2)
O4^xiv^—K4—O2^xvi^	77.97 (3)	Na2^vii^—Cl3—K4^xxii^	91.846 (11)
O3^xv^—K4—O2^xvi^	62.72 (3)	K1^i^—Cl3—K4^xxii^	88.154 (10)
O2^xiii^—K4—Cl1	77.20 (2)	K3^xi^—Cl3—K4^xxii^	88.576 (7)
O6^xii^—K4—Cl1	71.69 (2)	K3^xxv^—Cl3—K4^xxii^	91.195 (7)
O6^xi^—K4—Cl1	70.78 (2)	Na2^vii^—Cl3—K4^xix^	91.846 (10)
O4^xiv^—K4—Cl1	132.25 (2)	K1^i^—Cl3—K4^xix^	88.154 (10)
O3^xv^—K4—Cl1	125.74 (3)	K3^xi^—Cl3—K4^xix^	91.195 (7)
O2^xvi^—K4—Cl1	74.06 (2)	K3^xxv^—Cl3—K4^xix^	88.576 (7)
O2^xiii^—K4—Cl3^xiv^	75.23 (2)	K4^xxii^—Cl3—K4^xix^	176.31 (2)

Symmetry codes: (i) −*x*+1, −*y*+1, −*z*; (ii) *x*−1/2, *y*−1/2, −*z*; (iii) −*y*+1, *x*−1/2, −*z*; (iv) *y*−1/2, −*x*+1, −*z*; (v) *x*, *y*, *z*−1; (vi) *x*+1/2, *y*−1/2, −*z*+1; (vii) −*x*+1, −*y*+1, −*z*+1; (viii) *y*, −*x*+1/2, *z*; (ix) −*y*+3/2, *x*, *z*; (x) *x*+1/2, *y*−1/2, −*z*; (xi) −*y*+1/2, *x*, *z*; (xii) −*x*+1/2, −*y*+1/2, *z*; (xiii) −*y*+1, *x*−1/2, −*z*+1; (xiv) *y*−1/2, −*x*, −*z*+1; (xv) −*x*+1/2, −*y*+1/2, *z*+1; (xvi) *x*−1/2, *y*−1/2, −*z*+1; (xvii) *y*+1/2, −*x*+1, −*z*; (xviii) *x*+1/2, *y*+1/2, −*z*+1; (xix) *y*+1/2, −*x*+1, −*z*+1; (xx) −*x*+1/2, −*y*+1/2, *z*−1; (xxi) −*x*, −*y*+1, −*z*+1; (xxii) −*y*, *x*+1/2, −*z*+1; (xxiii) *x*−1/2, *y*+1/2, −*z*+1; (xxiv) *x*, *y*, *z*+1; (xxv) *y*, −*x*+3/2, *z*.
